# Evaluation of *Proctophyllodes huitzilopochtlii* on feathers from Anna’s (*Calypte anna*) and Black-chinned (*Archilochus alexandri*) Hummingbirds: Prevalence assessment and imaging analysis using light and tabletop scanning electron microscopy

**DOI:** 10.1371/journal.pone.0191323

**Published:** 2018-02-14

**Authors:** Youki K. Yamasaki, Emily E. Graves, Robin S. Houston, Barry M. OConnor, Patricia E. Kysar, Mary H. Straub, Janet E. Foley, Lisa A. Tell

**Affiliations:** 1 Department of Veterinary Microbiology and Pathology, Washington State University, Pullman, Washington, United States of America; 2 Department of Pathology, Microbiology, and Immunology, University of California, Davis, Davis, California, United States of America; 3 Department of Environmental Science and Policy, University of California, Davis, Davis, California, United States of America; 4 Department of Ecology and Evolutionary Biology, Museum of Zoology, University of Michigan, Ann Arbor, Michigan, United States of America; 5 Electron Microscopy Core Laboratory, Department of Cell Biology and Human Anatomy, University of California Davis Health System, University of California, Davis, Davis, California, United States of America; 6 Department of Medicine and Epidemiology, School of Veterinary Medicine, University of California, Davis, Davis, California, United States of America; Universidad de Granada, SPAIN

## Abstract

*Proctophyllodes huitzilopochtlii* Atyeo & Braasch 1966 (Acariformes: Astigmata: Proctophyllodidae), a feather mite, was found on feathers collected from five hummingbird species in California. This mite has not been previously documented on feathers from Anna’s (*Calypte anna* [Lesson 1829]) or Black-chinned (*Archilochus alexandri* [Bourcier & Mulsant 1846]) Hummingbirds. A total of 753 hummingbirds were evaluated for the presence of mites by species (Allen’s n = 112; Anna’s n = 500; Black-chinned n = 122; Rufous n = 18; Calliope n = 1), sex (males n = 421; females n = 329; 3 unidentified), and age (juvenile n = 199; after-hatch-year n = 549; 5 unidentified). Of these 753 hummingbirds evaluated, mites were present on the rectrices of 40.9% of the birds. Significantly more Anna’s Hummingbirds were positive for rectricial mites (59.2%) compared with 8.2% of Black-chinned, 0.9% of Allen’s, 5.6% of Rufous Hummingbirds, and 0% for Calliope (p-value < 0.0001). Across all hummingbird species, male hummingbirds (44.9%) had a higher prevalence of rectricial mites compared to female hummingbirds (36.2%; p-value = 0.004), while juvenile hummingbirds (46.2%) had a non-significantly higher prevalence compared to after-hatch-year hummingbirds (39.0%; p-value = 0.089). On average, the percentage of the long axis of the rachis occupied by mites for the outer rectrices (R4 and R5) was 19%, compared to 11% for inner rectrices (R1 and R2), a significant difference (p-value = <0.0001). There was a marginal lack of significance for symmetrical distribution of tail mites with the mean left side percentage of long axis of the rachis occupied by mites being 16% and very close to the mean right side score of 18% (p-value = 0.003). The identification of the feather mite species was based on light microscopic morphometry, and mite distribution on feathers was further evaluated using tabletop scanning electron microscopy (TSEM). The hummingbird–feather mite relationship is not well understood, but the specialized TSEM technique may be especially useful in examining natural positioning and developmental aspects of the mites since it allows *in situ* feather examination of live mites.

## Introduction

Evaluating host-mite relationships is important when studying wild bird populations. Most of the literature reports for mites found on hummingbirds have predominantly been systematics studies, while hummingbird-mite ecological relationships remain relatively understudied [1]. Sarcoptiform mites in the group Psoroptidia (Astigmata) have been reported from numerous birds and include mite species whose effects on the host range from pathological (e.g. scaly-leg and depluming mites, intranasal and air sac mites), moderately damaging (skin surface feeders) to generally commensal (most feather mites on vanes) or even potentially mutualistic (feather mites that may consume potential microbial or fungal pathogens) [2]. Given this wide range of host-mite relationships, studies evaluating hummingbird-mite interactions will further our knowledge base for these avian pollinators.

Hummingbirds are host to a diversity of feather mites, mainly in the family Proctophyllodidae; however, little is understood about the hummingbird-feather mite relationship. Most of the hummingbird feather mites are members of the diverse proctophyllodid tribe Rhamphocaulini in the subfamily Pterodectinae [3–10], with only one species in the subfamily Proctophyllodinae known to be associated with hummingbirds. *Proctophyllodes huitzilopochtlii* Atyeo & Braasch 1966 was described from thirteen species of hummingbirds from Mexico and Texas: *Amazilia beryllina* (Deppe 1830), *A*. *rutila* (Delattre 1843), *A*. *violiceps* (Gould 1859), *Chlorostilbon canivettii* (Lesson 1832), *Colibri thalassinus* (Swainson 1827), *Cynanthus latirostris* Swainson 1827, *C*. *sordidus* (Gould 1859), *Eugenes fulgens* (Swainson 1827), *Hylocharis leucotis* (Vieillot 1818), *Lampornis clemenciae* (Lesson 1829), *Selasphorus platycercus* (Swainson 1827), *S*. *rufus* (Gmelin 1788), and *S*. *sasin* (Lesson 1829) [11]. The same authors also reported specimens from an unidentified hummingbird from California [11]. This mite species was later reported from *Chlorostilbon lucidus* (Shaw 1812) [12] and *Amazilia fimbriata* (Gmelin 1788) [13] in Brazil, *Amazilia rutila* in El Salvador [11,14] and *Sephanoides sephanoides* (Lesson 1827) in Chile [15], greatly increasing the known geographical range of the mite on hummingbird hosts.

In the past, most feather mite reports have been based on specimens removed from feathers and examined by light microscopy; however, these works do not report the positioning of the various mite stages on the feathers *in situ*. Here we documented the presence of *P*. *huitzilopochtlii* on feathers from Anna’s (*Calypte anna* [Lesson 1829]), Black-chinned (*Archilochus alexandri* [Bourcier & Mulsant 1846]), Rufous (*Selasphorous rufus*), and Allen’s (*Selasphorous sasin*) Hummingbirds in California, identifying the mites based on light microscopy morphometric parameters and using table top scanning electron microscopy (TSEM) to describe mite distribution on the feathers.

## Methods

### Assessment of rectricial mite prevalence across hummingbird species, age and sex classes and mite distribution along the rachial long axis for individual rectrices

During March to October, 2015 and February to October, 2016, hummingbirds were trapped using feeder Hall traps [16] at four primary site urban yard locations in California (locations are approximate in order to protect privacy of land owners): Winters (38°31′49″N, 121°51′2″W), Inverness (38° 4' 36'' N, 122° 49' 52'' W), Placerville (38°44'36'' N, 120°56'6'' W), and Avalon (33° 20' 23", 118° 19' 52" W). The birds were aged and sexed, and hummingbird species identified using published methods [17,18]. Since mites were predominantly found on the rectrices, this portion of the study was limited to these tail feathers.

Mites on feathers were visually evaluated with the naked eye for all captured hummingbirds, and presence or absence data were used to estimate prevalence. The observers used their fingers to fan the rectrices so that the individual tail feathers could be observed without any overlap of neighboring feathers. The ventral or dorsal location of the mites on the feathers was recorded.

For all statistical tests, a p-value of less than 0.05 was considered significant. Differences across species, sex, and age class in prevalence were evaluated using chi-square contingency tests. Mite distribution along the long axis of the rachis for each rectrix was evaluated for a subset (n = 184 birds) of captured Anna’s Hummingbirds. The scoring system ranged from 0–100% and mite occupation was scored in 10% increments along the rachis unless 5 or fewer mites (recorded as 5%) or a single mite (recorded as 1%) were observed along the rachis. Differences among male and female hummingbirds in percent of rachis length occupied by mites were assessed with a Student’s t-test. We assessed the *a priori* hypotheses that inner vs. outer retrices and left vs. right would differ in their scores for length of the rachis that was occupied by mites using paired t-tests.

### Collection of mites for species identification

For this portion of the study, hummingbirds were captured and identified as previously described [17,18]. All rectrices (5/side) and remiges (10/wing) were visually assessed for the presence of feather mites, selected feathers with mites were pulled for sampling, and birds were released. Using digital traction, a feather (single rectrix and/or remex) was carefully removed at the feather base near the dermal attachment. In most cases, a single rectrix was removed from each bird, but in rare cases (n = 2 birds) both a rectrix and remex were removed. Feathers were collected from after-hatch-year male (n = 16), after-hatch-year female (n = 9), hatch-year male (n = 2) or hatch-year female (n = 3) Anna’s Hummingbirds (ANHU) and after-hatch-year male (n = 3) and after-hatch-year female (n = 2) Black-chinned Hummingbirds (BCHU). For comparison purposes, mite specimens were also collected from feathers from after-hatch-year male Rufous (n = 1) and Allen’s (n = 1) Hummingbirds for which the presence of the mite has been previously reported [11].

For TSEM assessment of mites, individual feathers were removed from birds in the field, transported in 6-well cell culture plates to the laboratory, and observed within 4 hours of sampling. The majority of feathers inhabited by mites were rectrices with the exception of two birds (a hatch-year female and an after-hatch-year female Anna’s Hummingbird) that had mites on both rectrices and remiges, both of which were collected. For light microscopic morphometric examination, feathers with mites were placed in cryogenic vials containing 70% ethanol. All methods associated with this study were approved by the UC Davis Institutional Animal Care and Use Committee, and permits (LAT) were granted by the United States Geological Service Bird Banding Laboratory, United States Federal Fish and Wildlife Service, and the California Department of Fish and Wildlife.

### Light microscopic morphometric analysis

Nymphs and adult male and female mites were removed from the feathers with a fine probe and mounted in Hoyer’s medium on microscope slides. After drying on a slide warmer for 48 hr, the mites had cleared sufficiently such that dimensional measurements could be taken of male and female adults, as well as male lamellae using a calibrated ocular micrometer mounted in a compound light microscope (Olympus CX40 Microscope: Olympus Optical Company, Ltd., Tokyo, Japan). Dimensions taken were based on those reported in original descriptions of mites [11], i.e. for males, the length was measured from the pedipalp apex to the internal postanal setae; for females the length was measured from the pedipalp apex to the posterior extremity of hysterosomal lobes excluding appendages. Width was measured at the widest point behind the second pair of legs for both sexes.

Mite specimens from our study were compared with some of the original material in the collection of W.T. Atyeo. This original material was used by Atyeo and Braasch in the original description of *P*. *huitzilopochtlii* [11]. This collection is now housed in the University of Michigan Museum of Zoology, Ann Arbor, MI and was, thus available to one of the co-authors (BMOC) for comparison. Additionally, the Atyeo collection includes one male and one female identified as *P*. *huitzilopochtlii* by Atyeo from *Calypte anna* (NU1601) with collection data: California: Santa Cruz Co., Santa Cruz, IV-1937, C.D. Streator. These may be the specimens mentioned from California without host identification in the original work [11].

### Tabletop scanning electron microscopic analysis

Unfixed, recently removed feathers from hummingbirds were mounted on aluminum stubs using double stick carbon conductive tabs (Carbon Conductive Tabs for SEM: Ted Pella, Inc., Redding, CA) for scanning electron microscopy. The dorsal aspect of the feather was gently pressed onto the tabs and the uncoated mounted feather sample was immediately placed into a tabletop scanning electron microscope (SEM; Tabletop SEM Microscope: Hitachi TM-3030Plus, Hitachi America, Ltd, Tarrytown, NY). The mites were imaged live using 15kV in mixed signal mode (combined secondary and backscatter mode). Using the TSEM measuring tool function, measurements were taken for individual mites to evaluate if TSEM values were comparable to light microscopic mite measurements for specimens mounted on glass slides. In addition, TSEM measurements were taken from individual mites from both host hummingbird species to assess if there were differences in standard measurements between mite populations from the two host species.

### DNA sequencing

Mites from Anna’s Hummingbirds were removed from feathers stored in ethanol and placed into open, individual Eppendorf tubes and allowed to dry overnight at 25°C. DNA was extracted from individual mites using a commercial kit (Qiagen QIAamp DNA Micro Kit: Qiagen Inc., Valencia, CA). An additional pre-incubation step was added to the manufacturer’s protocol in which mites were incubated overnight at 56°C in 180μL Buffer ATL in an orbital shaker at 26.2 rad/s. A 644-bp region of the cytochrome C oxidase I (COI) gene was amplified by PCR using primers bcdF05 and bcdR04 [19]. Amplification conditions were modified slightly from the original reference: a reaction volume of 50μL was used and the number of amplification steps was increased from 40 to 45. DNA bands were extracted using a commercial kit (QIAquik Gel Extraction Kit: Qiagen Inc., Valencia, CA) according to manufacturer’s instructions. DNA was sequenced at a University of California, Davis laboratory (^f^DNA Sequencing Facility, Davis, CA) using the forward primer. Sequences were archived with GenBank (Accession #s MF802486 and MF802487).

## Results

### Prevalence of rectricial mites by hummingbird species, age, and sex classes

A total of 753 hummingbirds was evaluated for the presence of tail mites (Allen’s n = 112; Anna’s n = 500; Black-chinned n = 122; Rufous n = 18; Calliope n = 1). The overall prevalence of rectricial mites across all hummingbird species examined was 40.9% (n = 308). Anna’s Hummingbirds (59.2%) had a significantly higher prevalence than Black-chinned (8.2%), Allen’s (0.9%), Calliope (0%) or Rufous (5.6%) Hummingbirds (X-squared = 207.4044, df = 4, p-value = <0.0001). Across all hummingbird species, males tended to have a higher prevalence of rectricial mites (44.9% of n = 421) than females (36.2% of n = 329) (X-squared = 8.2368, df = 1, p-value = 0.004) and juveniles tended to have higher prevalence (46.2% of n = 199) than after-hatch-years (39.0% of n = 549), although the difference was not significant (X-squared = 2.884, df = 1, p-value = 0.089). Table 1 shows the mite prevalence of rectricial mites by hummingbird species, age, and sex.

**Table 1:** *Proctophyllodes huitzilopochtlii* prevalence (% of individual hummingbirds examined that were positive for presence of rectricial mites) by hummingbird species, age, and sex. The prevalence was calculated as the number of individual birds with tail mites divided by the total number of individuals examined.

Factor	Prevalence	Number of Individuals Evaluated	95% Confidence Interval	p-value
***Species***				2.2e-16
Allen’s Hummingbird *(Selasphorus sasin)*	0.9	112	0.0005–0.06	
Anna’s Hummingbird *(Calypte anna*)	59.2	500	0.55–0.64	
Black-chinned Hummingbird (*Archilocus alexandri*)	8.2	122	0.04–0.15	
Rufous Hummingbird (*Selasphorus rufus*)	5.6	18	0.003–0.29	
Calliope Hummingbird (*Selasphorus calliope*)	0	1	NA	
***Sex***				0.004
Male	44.9	421	0.40–0.50	
Female	36.2	329	0.31–0.41	
***Age***				0.089
Juvenile	46.2	199	0.39–0.53	
After-Hatch-Year	39.0	549	0.35–0.43	

### Mite distribution along the rachial long axis for individual rectrices (Anna’s hummingbirds)

Of 184 Anna’s Hummingbirds for which percentage of rachis length with mites present could be evaluated, 21 had at least one molting retrix and were not included in the following analysis. During evaluation of the birds’ rectrices, a notably high load of mites along ventral surfaces of outer retrices (R4 and R5) was observed, so we sought to determine whether there was a significant difference between inner and outer retrices. Among 163 Anna’s Hummingbirds, on average, the percentage of outer rachis length (R4 and R5) with mites present was 19%, compared to 11% for inner rectrices (R1 and R2), a significant difference (t = -5.3359, df = 161, p-value = < 0.0001, Table 2). Among the Anna’s Hummingbirds’ tail feathers, there was weakly significant lack of symmetry of infestation, with mean left side scores of 16% and right side scores of 18% (t = -3.0532, df = 161, p-value = 0.003). There were no significant differences in percentages of rachis length with mites present for individual retrices between male and female Anna’s Hummingbirds (*p* = 0.76).

**Table 2:** Average (± SD) percentage of the long axis of the feather rachis with *Proctophyllodes huitzilopochtlii* present for each tail feather (rectrices 1–5, right and left sides. Results are reported for sex of Anna’s Hummingbirds (n = 98 males and 65 females) that had mites present on any rectrix. The percentage scale ranged from 0–100% in 10% increments unless 5 or fewer mites were present (reported as 5%) or a single mite was present (reported as 1%). Not all rectrices had mites present. “LR” denotes “left rectrix” and “RR” denotes “right rectrix.” All mites were observed on the ventral aspect of the feathers.

Rectrix	Anna’s Hummingbirds *(Calypte anna)*	
	Male (n = 98)	Female (n = 65)
**RR1**	*5*.*4%* (SD = 3.6) Range: 0%-70%	*4*.*6%* (SD = 9.7) Range: 0%-50%
**LR1**	*6*.*5%* (SD = 14.8) Range: 0%-70%	*3*.*4%* (SD = 8.2) Range: 0%-40%
**RR2**	*20*.*6%* (SD = 23.3) Range:0%-80%	*14*.*1%* (SD = 19.0) Range: 0%-70%
**LR2**	*17*.*9%* (SD = 22.0) Range: 0%-80%	*11*.*9%* (SD = 15.7) Range: 0%-50%
**RR3**	*30*.*4%* (SD = 23.7) Range: 0%-90%	*20*.*7%* (SD = 22.8) Range: 0%-80%
**LR3**	*28*.*8%* (SD = 24.2) Range: 0%-90%	*18*.*4%* (SD = 20.9) Range: 0%-70%
**RR4**	*27*.*8%* (SD = 24.4) Range: 0%-90%	*19*.*3%* (SD = 22.5) Range: 0%-90%
**LR4**	*24*.*2%* (SD = 23.6) Range: 0%-90%	*16*.*9%* (SD = 21.7) Range: 0%-80%
**RR5**	*12*.*1%* (SD = 16.8) Range: 0%-80%	*21*.*3%* (SD = 26.1) Range: 0%-90%
**LR5**	*13*.*1%* (SD = 18.7) Range: 0%-80%	*17*.*0%* (SD = 22.8) Range: 0%-80%

### Light microscopic morphometric analysis

**Fig 1:**
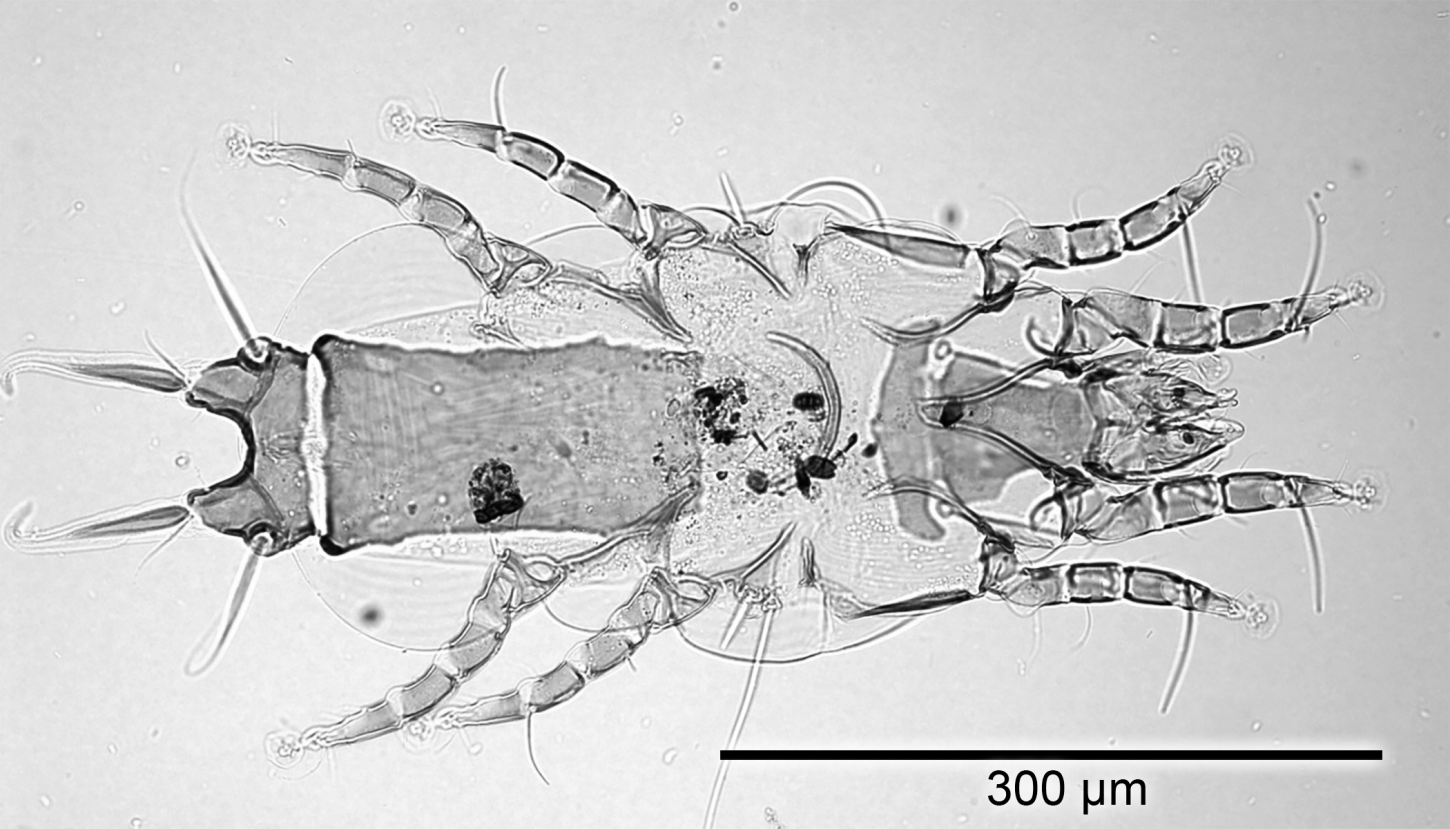
Light microscopy photomicrograph of an adult female *Proctophyllodes huitzilopochtlii* from a rectrix of an Anna’s hummingbird.

**Fig 2:**
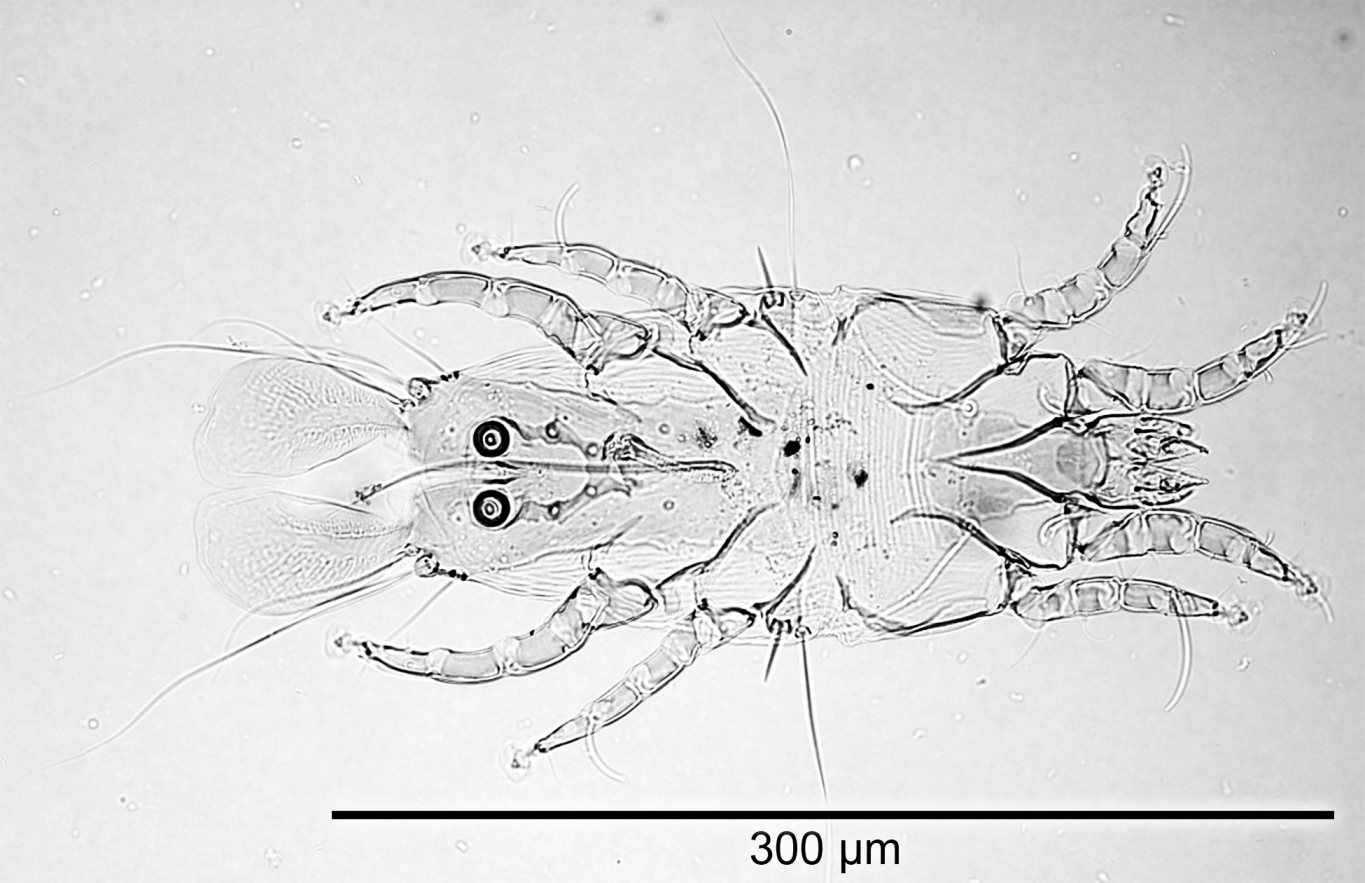
Light microscopy photomicrograph of an adult male *Proctophyllodes huitzilopochtlii* from a rectrix of an Anna’s hummingbird.

Dimensions for male and female specimens of *P*. *huitzilopochtlii* are recorded in Table 3. The identities of female (Fig 1) and male (Fig 2) mites from both Anna’s and Black-chinned Hummingbirds from this study were confirmed as *P*. *huitzilopochtlii* as their measurements were comparable with the original specimens described as *P*. *huitzilopochtlii*, including paratypes from *Lampornis clemenciae* and specimens from all other hosts reported. Although the Atyeo and Braasch [11] taxonomic key in that work stated male lamellae length (“over 130 μ”) on page 36 as characteristic of *P*. *huitzilopochtlii*, in the actual description of the species by Atyeo and Braasch [11] a measurement for the same structure was given as “96 μ” on page 50, more in line with our measurements by light microscopy.

**Table 3:** Average ± SD (mite numbers) anatomic measurements of adult male and female *Proctophyllodes huitzilopochtlii* taken from feathers of Anna’s (*Calypte anna)* and Black-chinned (*Archilochus alexandri)* hummingbirds using light (LM) and tabletop scanning electron (TSEM) microscopic imaging.

Hummingbird Host species	Male length, excluding lamella (μm)		Male width (μm)		Male lamella length (μm)		Female length (μm)		Female width (μm)	
	LM	TSEM	LM	TSEM	LM	TSEM	LM	TSEM	LM	TSEM
**Anna’s Hummingbird *(Calypte anna)***	325 ± 25.0 (23)	304 ± 13.2 (17)	141 ± 25.2 (23)	117 ± 5.5 (25)	93.9 ± 5.7 (23)	85 ± 8.6 (29)	462 ± 19.1 (58)	415 ± 20.4 (37)	179 ±48.4 (58)	142 ± 9.4 (36)
***Black-chinned Hummingbird (Archilochus alexandri)***	319 ± 18.6 (9)	285 ± 6.2 (3)	132 ± 6.6 (9)	109 ± 4.9 (3)	75 ± 11.5 (9)	62.1 ± 6.8 (3)	459 ± 18.7 (15)	394, 418 (2)*	165 ± 10.4 (15)	126, 146 (2)*

*Only two mites were available for measuring, therefore data for individual mites reported.

### Tabletop scanning electron microscopic analysis

Overall, mite numbers were fewer per feather for Black-chinned versus Anna’s Hummingbirds. The highest and lowest mite numbers that could be measured (proper positioning and not overlap with other mites) ranged from 29 adult female mites on a single feather from an Anna’s Hummingbird compared to 2 female mites from a Black-chinned Hummingbird and 24 adult male mites on a single feather from an Anna’s Hummingbird compared to one adult male mite from a Black-chinned Hummingbird. For the feathers from the Black-chinned Hummingbirds, most of the mites were located relatively distant from the rachis. For Anna’s Hummingbird feather samples, mites were found between barbs in proximity to the main shaft. Mites moved around on the feather after being refrigerated (4° C) for 24 hours, and after extended refrigeration time (greater than a week), the mites started to shrivel and implode. No evidence of structural damage was observed on any of the feathers.

Average anatomic measurements of adult male and female *P*. *huitzilopochtlii* mites from Anna’s and Black-chinned Hummingbirds using TSEM imaging are reported in Table 3. Compared to anatomic measurements obtained using light microscopic imaging, the TSEM measurements were consistently less for all measurements.

**Fig 3:**
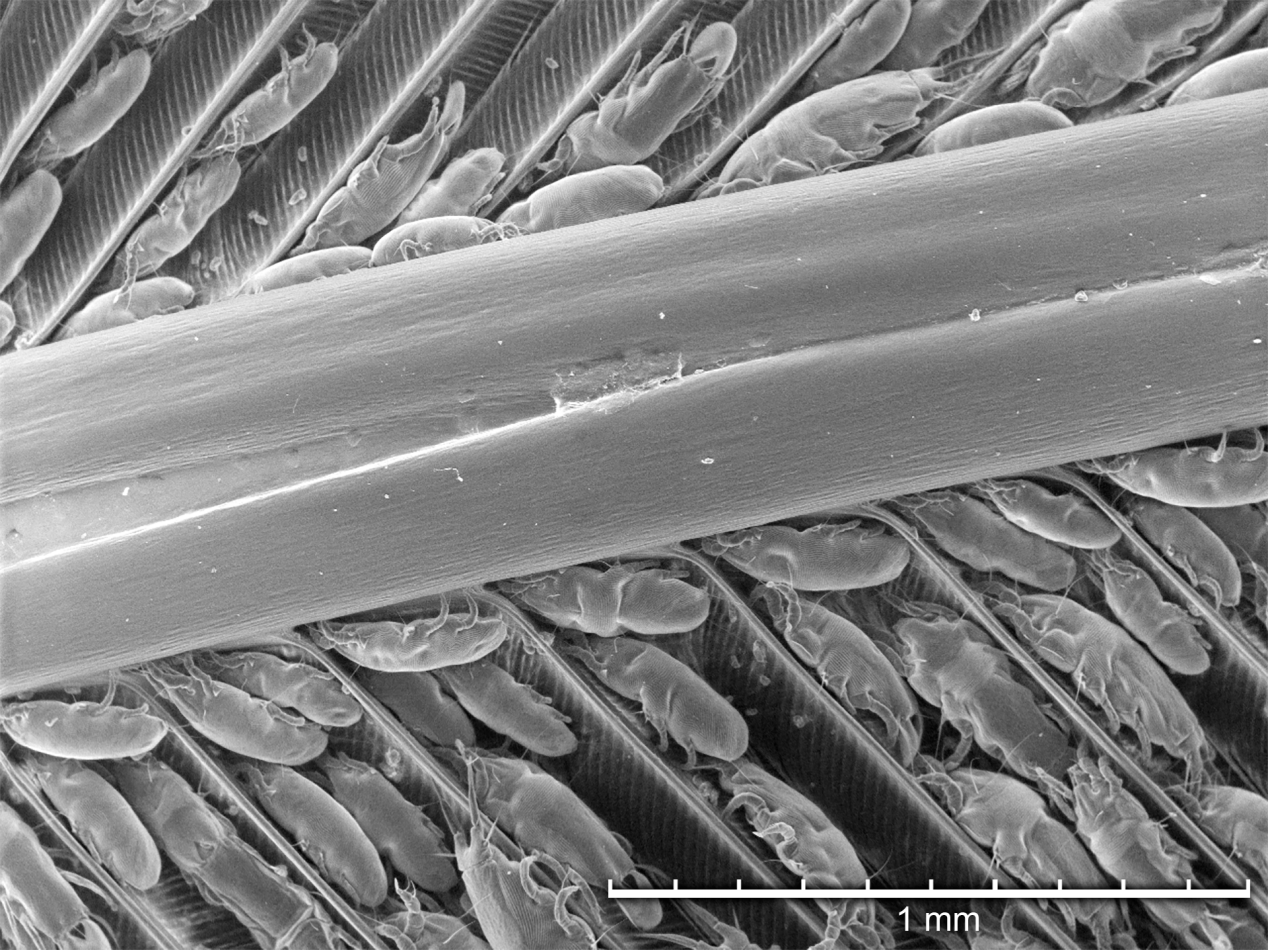
Tabletop scanning electron photomicrograph of *Proctophyllodes huitzilopochtlii* mites along the main shaft of a rectrix from an Anna’s hummingbird.

**Fig 4:**
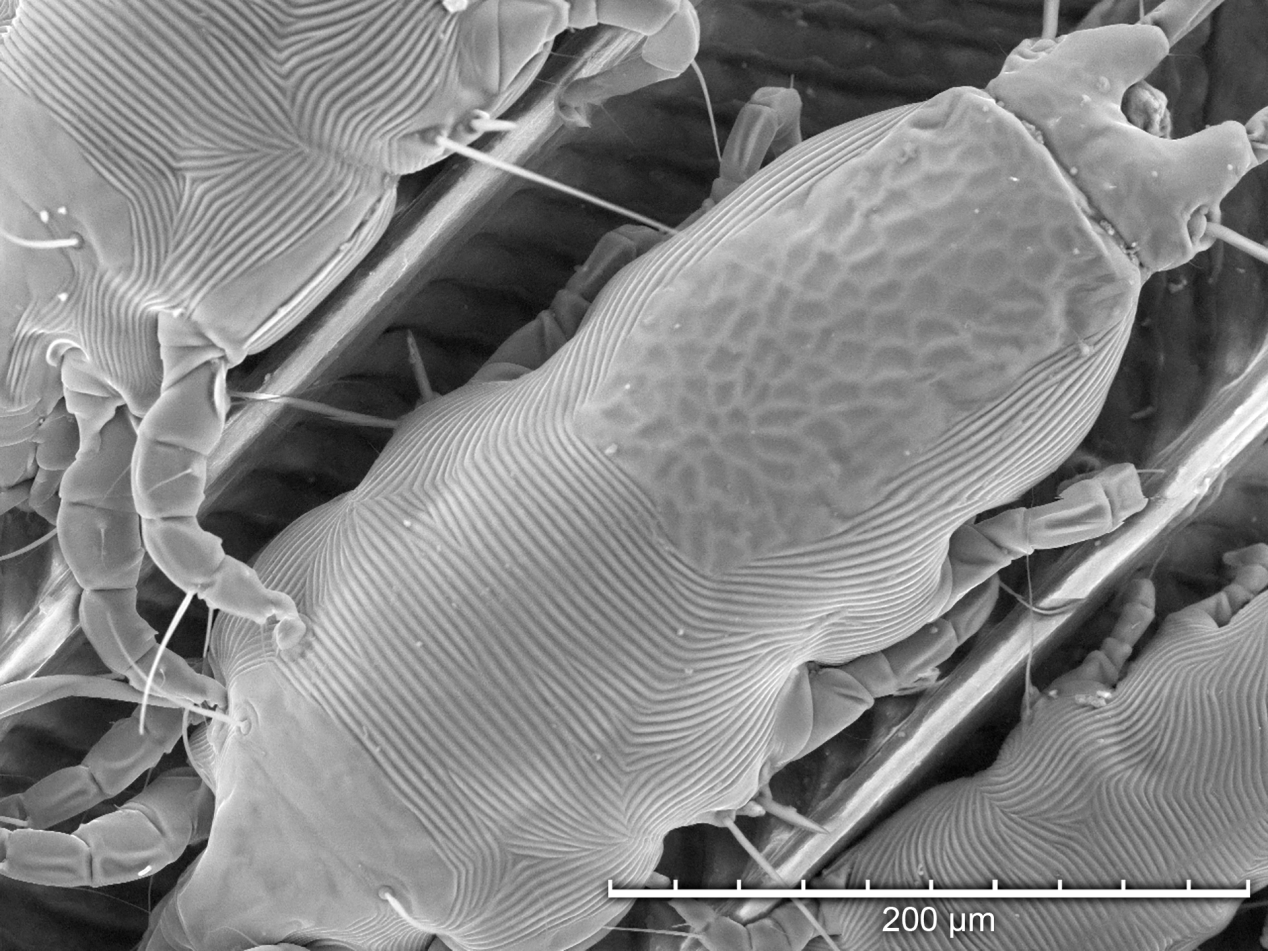
Tabletop scanning electron photomicrographs showing magnified detail of the hysterosomal shield a female *Proctophyllodes huitzilopochtlii*.

**Fig 5:**
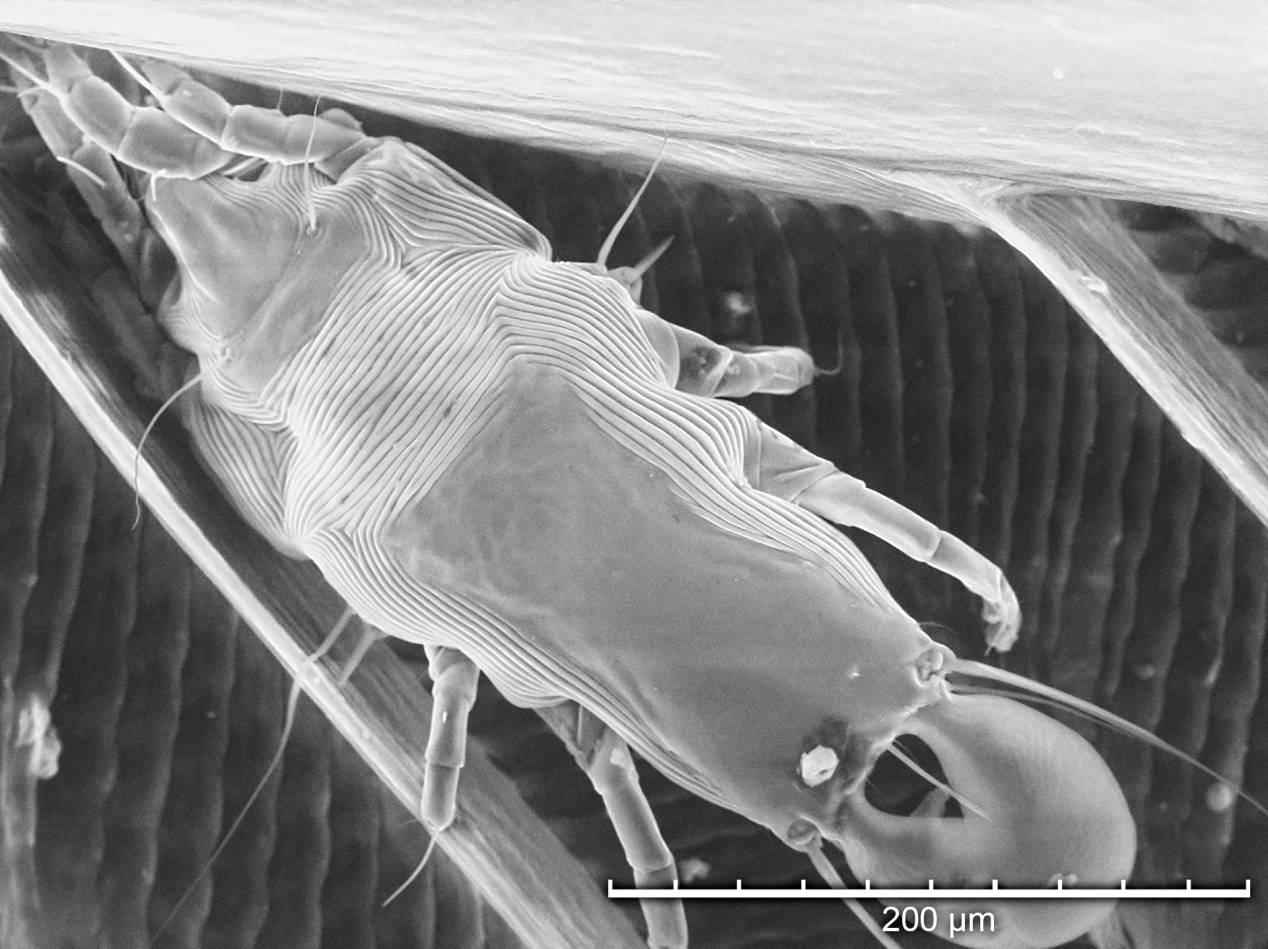
Tabletop scanning electron photomicrograph of an adult male *Proctophyllodes huitzilopochtlii* on a rectrix from Black-chinned hummingbird.

**Fig 6:**
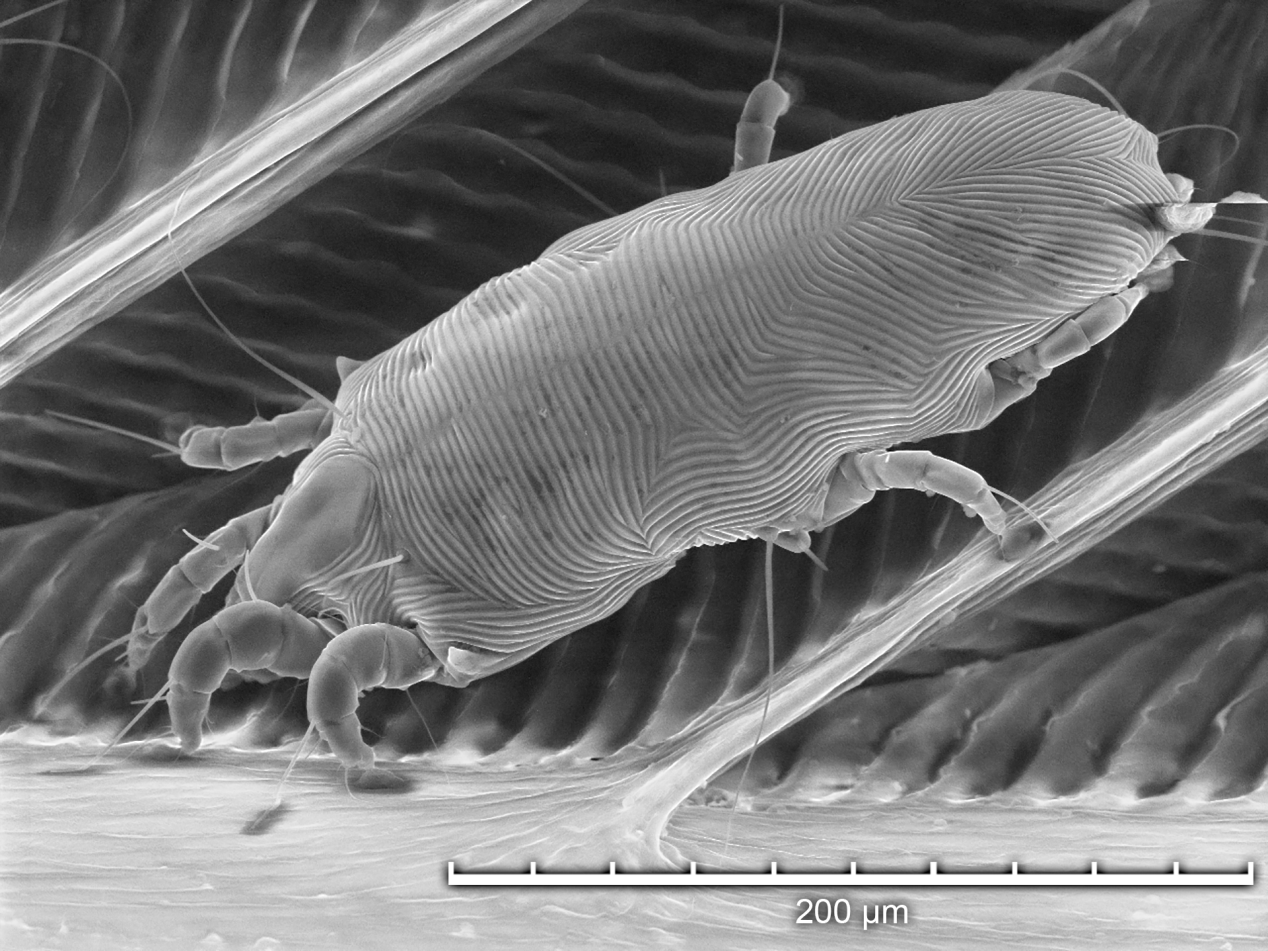
Tabletop scanning electron photomicrograph of a male protonymph *Proctophyllodes huitzilopochtlii* on a rectrix from an Anna’s hummingbird.

**Fig 7:**
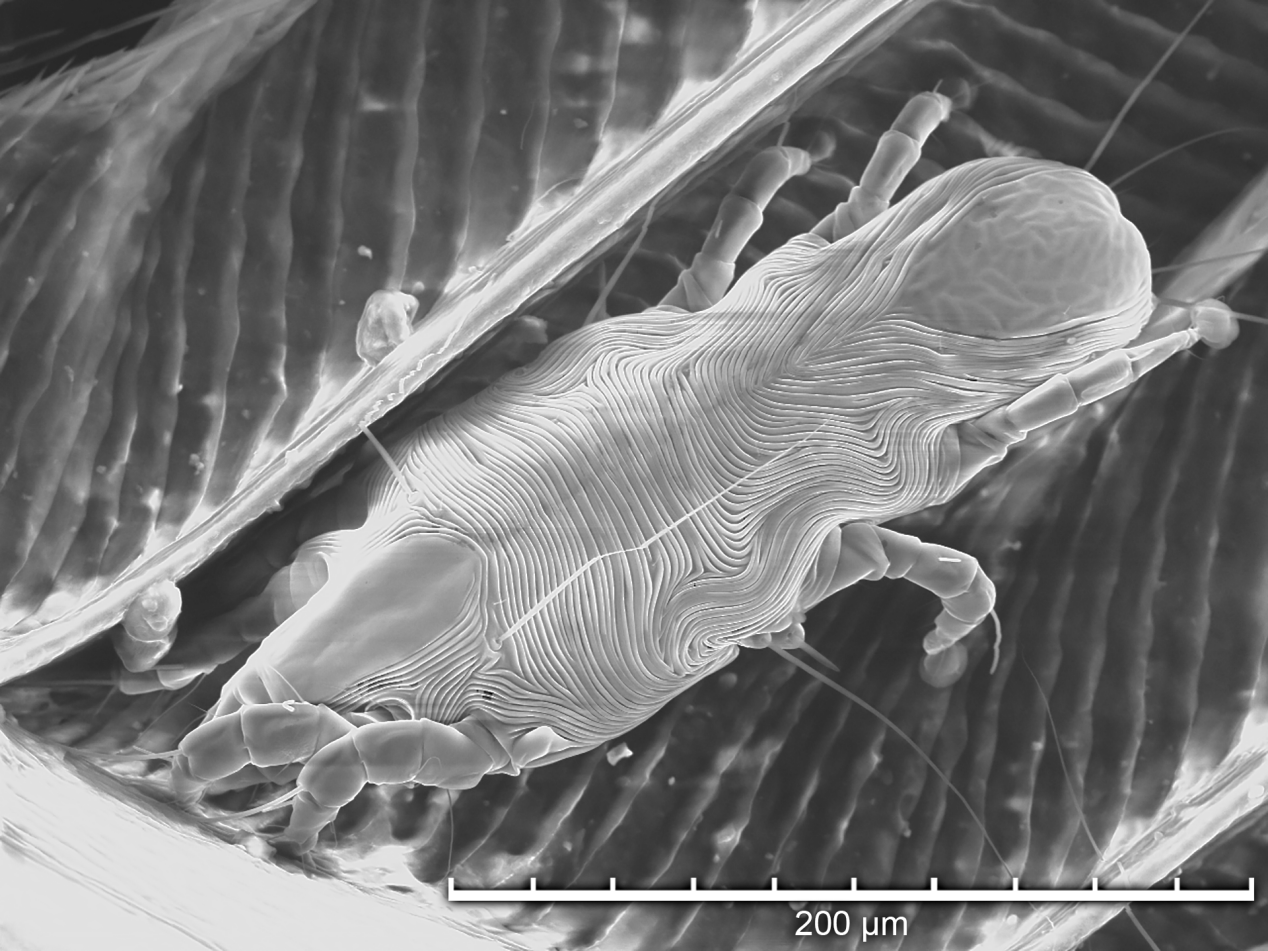
Tabletop scanning electron photomicrograph of female tritonymph *Proctophyllodes huitzilopochtlii* on a rectrix from an Anna’s hummingbird.

**Fig 8:**
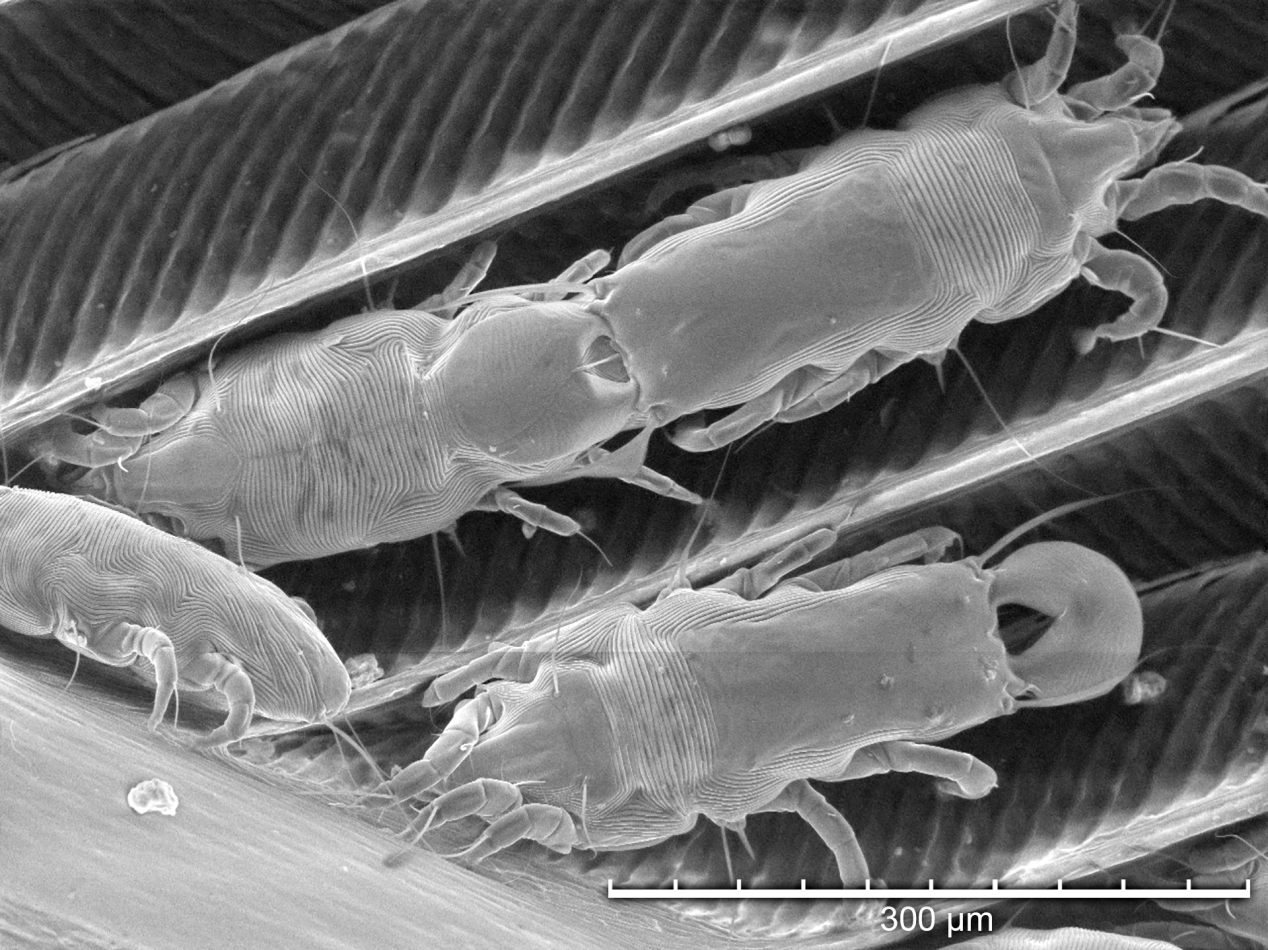
Tabletop scanning electron photomicrograph of *in situ Proctophyllodes huitzilopochtlii* male and female nymphs in a pre-copulatory guarding posture (PCGP) where a distinct medial ecdysial line is evident.

Using TSEM, all developmental stages were observed except larvae (Fig 3). Adult females (Fig 4) were more numerous than adult males (Fig 5), and both protonymphs (Fig 6) and tritonymphs (Fig 7) were present. The juvenile mites were found closest to the rachis (Fig 3) and oriented with the anterior end toward the proximal aspect of the feather. Typical pre-copulatory guarding posture was observed, where the male mite was attached to a female nymph (Fig 8). In many species of *Proctophyllodes*, protonymphs and tritonymphs exhibit sexual dimorphism, with female nymphs often bearing a pair of dorsal projections near the posterior end of the hysterosoma. In *P*. *huitzilopochtlii*, projections are absent, but female nymphs possess a sclerite on the posterior hysterosoma (Fig 7) that is absent in male nymphs (Fig 6). The sclerite is similar to that of the adult female (Fig 4), but smaller, and is positioned such that it may contact the male para-anal suckers during juvenile guarding and adult mating. Pre-copulatory guarding can be distinguished from actual mating pairs because the pre-copulatory nymph has reduced hysterosomal sclerotization and presents a distinct medial ecdysial line (Fig 8).

**Fig 9:**
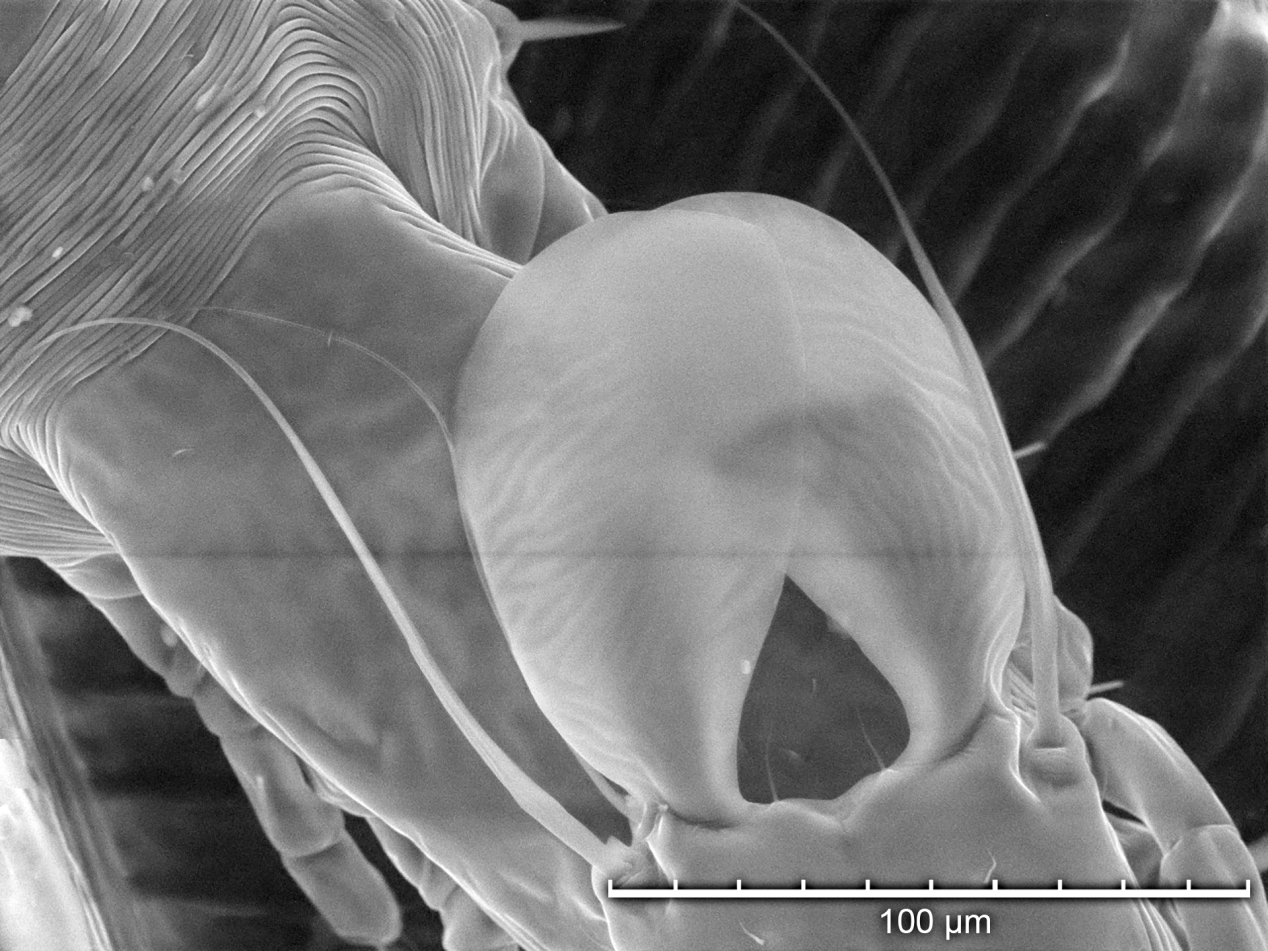
Tabletop scanning electron photomicrograph showing magnified detail of a *Proctophyllodes huitzilopochtlii* male lamellae oriented over an adult female’s posterior extremity.

**Fig 10:**
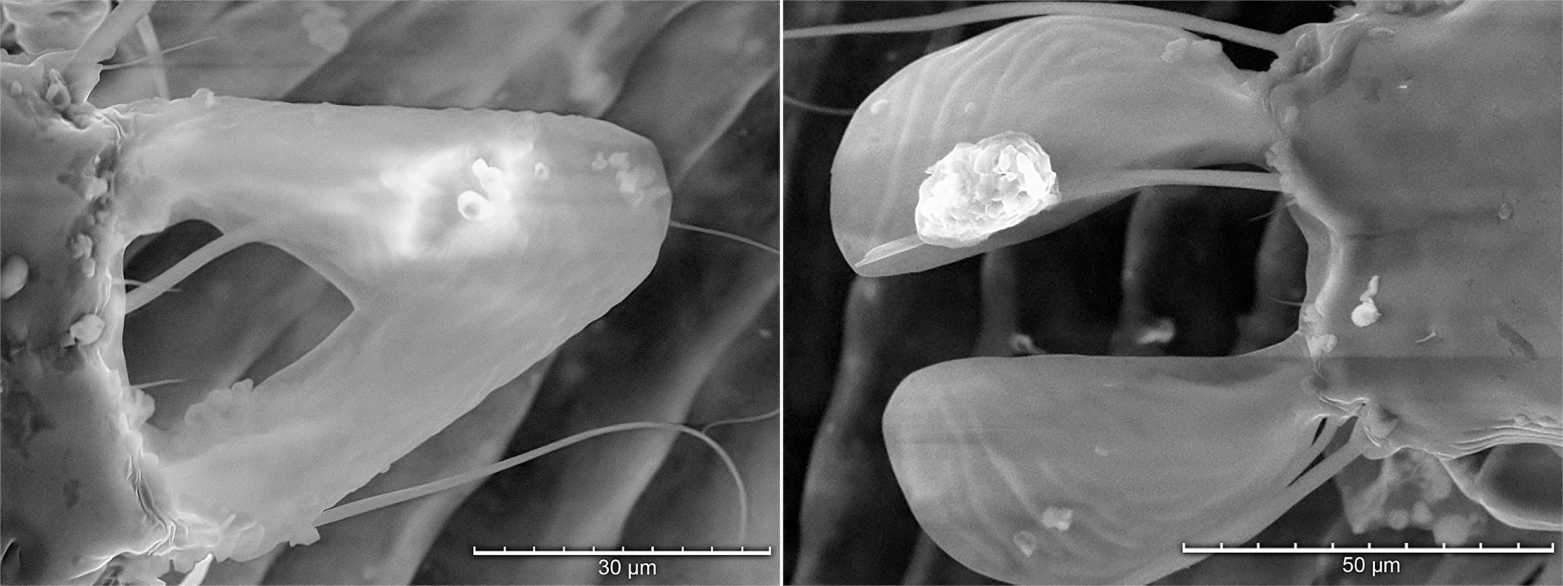
Tabletop scanning electron photomicrograph showing overlapping (A) or widely spread (B) lamellae of a male *Proctophyllodes huitzilopochtlii*.

TSEM provided detailed visualization of mite anatomic structures and variation within the same anatomic structure for live mites. Fig 9 shows that the overlapping lamellae provide a single contact surface with the female during pre-copulatory guarding and mating. Fig 10A and 10B show the degree of shape variation for the lamellae of *P*. *huitzilopochtlii*. In most male *Proctophyllodes* spp., each of the terminal lamellae is more or less symmetrical and do not overlap. However, in *P*. *huitzilopochtlii*, each lamella is asymmetrical, curving inward and usually overlapping the other distally (but see Fig 10B where the lamellae are non-overlapping).

## Discussion

To the authors’ knowledge, this study is the first to provide detailed prevalence and feather distribution information and live imaging of *Proctophyllodes huitzilopochtlii* on feather samples from both Anna’s and Black-chinned Hummingbirds. *P*. *huitzilopochtlii* has been reported from other hummingbird species that reside in (Allen’s Hummingbirds) or migrate through (Allen’s and Rufous Hummingbirds) California. However, no published studies have reported prevalence or mite distribution on feathers from Anna’s and Black-chinned Hummingbirds. The prevalence for Anna’s Hummingbirds with rectricial mites (58.9%) was notably higher compared to the other hummingbird species evaluated (0.89%-8.2%). In the locations surveyed for this study, Anna’s Hummingbirds seasonally co-reside with Allen’s and Black-chinned Hummingbirds. Rufous Hummingbirds can also be found, but only transiently as they pass through during migration. Therefore, multiple hummingbird species can be observed feeding at the same artificial feeders for part of the year. Since Anna’s hummingbirds are expanding their range [20] and could co-reside in habitats with other hummingbird species, understanding mite prevalence trends for *P*. *huitzilopochtlii* could potentially help predict whether other, potentially non- parasitic or parasitic, symbionts could move from Anna's Hummingbirds to other hummingbird species.

Besides differences in mite prevalence among, there was a higher mite prevalence for male than female hummingbirds. For our study, mite presence on feathers was evaluated during times when female birds were both off and on nests with chicks, therefore it is possible that this sex difference in mite prevalence could have been attributed to vertical transmission of feather mites to juvenile birds [21,22]. Unfortunately, due to our limited data set, mite prevalence based on hummingbird sex and nesting female hummingbirds was not possible to evaluate. However, these findings of lower mite prevalence on female relative to male hummingbirds and a higher prevalence in juvenile hummingbirds are consistent with previous reports. [21,22]

*P*. *huitzilopochtlii* was only observed on the ventral aspect of hummingbird tail feathers, and the percentage of the rachial long axis with mites present varied from a single mite up to 90% of the rachis length inhabited by mites. There was a higher percentage of mites on the lateral rectrices (R4 and R5) compared to the inner rectrices (R1 and R2). A study of mites on the blackcap, *Sylvia atricapilla*, (Sylviidae, [Linnaeus 1758]) in Spain reported that mite infestations can be symmetrical for left and right wing feathers [23]. For our study, there was a small and weakly significant difference between presence of mites on the left versus right sides (16 and 18%, respectively). Although not a parameter quantified in this study, two of the authors (EG and LT) observed that in some cases, there were up to three layers of mites lateral to the long axis of rachis, but a single layer of mites was the most predominant pattern.

Light microscopic evaluation of the mites for this study was sufficient to determine the genus and species. Sequences for the barcoding region of the COI gene for two *P*. *huitzilpochtlii* specimens from this study were also archived and might prove useful for future molecular diagnostics and/or assessment of phylogenetic relationships among closely related species of *Proctophyllodes*. *Proctophyllodes huitzilopochtlii* was originally placed in the *glandarinus* species group on the sole basis of the elongated male aedeagus [11]. Recognizing that group's artificiality, Gaud and Fain [24] and Mironov and Kopij [25] proposed two additional species groups (*mecistocaulus* and *caulifer*, respectively) for species previously assigned to that group. In the checklist of species of *Proctophyllodes* provided by Mironov [26], *P*. *huitzilopochtlii* was assigned to the *caulifer* group on the basis of the elongate aedeagus and lack of reniform accessory glands in the male. 19 species were assigned to the *caulifer* group, including 14 from the Old World and only 5 from the New World. Of the latter, three are associated with Icteridae (one of these, *P*. *longiphyllus* also with Cardinalidae), one with Vireonidae, and only *P*. *huitzilopochtlii* from the non-passeriform Trochilidae. Although the *caulifer* group may itself be artificial, being diagnosed only by the elongated aedeagus and the ancestral lack of reniform accessory glands, the species from Icteridae and Vireonidae are all characterized by very narrow posterior lamellae in the males and may, therefore form a natural group. The lamellae in *P*. *huitzilopochtlii* are straight and narrow towards the tip, another unusual state in *Proctophyllodes*. Looking forward, in order to determine the passerine bird host group from which *P*. *huitzilpochtlii*’s ancestor originally colonized hummingbirds, detailed molecular analysis with multiple genetic markers, as has been recently described by Klimov et al. [27] for other *Proctophyllodes* species, will be necessary.

TSEM imaging had several advantages over light microscopy for visualizing mites on feathers and measuring morphometric structures of *P*. *huitzilopochtlii* specimens. Using TSEM, it was possible to measure anatomic structures to compare specimens from different hosts, and multiple mites could be measured at once if they were in the correct position (i.e. flat and with all anatomic structures visible). Using the TSEM to measure mite anatomic structures substantially reduced time and effort, as no sample preparation was required, and multiple mite specimens could be measured on one feather. One thing to note was that mite anatomical measurements taken using TSEM were consistently smaller in comparison to those using light microscopic imaging. It is likely that when the mites were prepared for light microscopy, the weight of the mounting medium plus cover slip flattened the mite specimens, causing an artifact in excess of what the measurements taken on three-dimensional specimens by TSEM.

TSEM also had the added benefit of allowing observations on the position and orientation of live mites on the feather and allowed assessment of mite movement. Even though the mite movement seen in our study could not be directly attributed to cold storage, seasonal and ambient air temperature have been reported to influence the distribution of mites on feathers of blue tits [28,29]. In such cases, TSEM might allow for an even more granular evaluation of mite distribution on individual feathers themselves. Another advantage that we found with the TSEM was that it allowed imaging of live, untreated and non-conductive specimens. Earlier, we had tried using conventional SEM methods for sample preparation, but the mite morphology and location on the feather were altered. The TSEM methodology offered the advantage of documenting live mite positioning on the feather, shortly after the feather was sampled from the host. In addition, the samples did not need to be desiccated, therefore the mites’ three-dimensional physical characteristics could be preserved, and mite location could be recorded as found on the feather. Feather damage was not observed in our study, emphasizing the utility of TSEM for studying mite-host interactions and the opportunity for investigators to directly observe absence or presence of feather damage on a microscopic scale. Given the size [330(W)x617(D)x549(H) cm], weight (52 kg (MD+UVD) and power requirements (110V, 5 Amp, standard wall socket) of the TSEM unit, there is also the possibility for using it in the field to observe live mite specimens, an option that has not been possible for investigators previously.

The impact of feather mites on Anna’s and Black-chinned Hummingbirds requires future study. Relationships between most feather mites and birds still remain to be elucidated, but a commensalistic or even mutualistic interaction has been documented [30]. In our study, no feather damage by mites was observed, and it was unclear as to how the mites survive. Particulate matter was observed in the alimentary tracts of mites prepared for light microscopic analysis. This particulate matter included fungal spores [31,32] and other amorphous material that could include pollen [31]. Proctor’s personal observation was that pollen is often seen in alimentary tract of mites present on nectivorous birds [33]. For some mite species, uropygial gland oil is their main food source [34]. Even though no feather damage was observed, the abundance of mites found on the feathers of Anna’s Hummingbirds suggests a potential physical fitness cost such as stress on flight capabilities, or reproductive fitness such as mate attraction [35]. Sound production made during the courtship display dive of Anna’s Hummingbirds depends on the fourth and fifth rectrices (the most lateral tail feathers) [36], thus would be of interest. Chris Clark (University of California, Riverside, pers. comm.) suggests that over 50% of the width of the fourth and/or fifth rectrix would likely need to be inhabited by mites in order to impact sound production from the fifth tail feather, but this theory still needs to be evaluated. Additionally, even though it is not known how or when mites move from feather to feather with respect to molt in hummingbirds, it is generally accepted that mites avoid feathers that are about to molt and their distribution is adjusted accordingly [37,38,39]. For our study, partially sheathed incoming feathers were occasionally observed to have mites (on the non-sheathed portion of the feather), so further study of how molt affects mite distribution on feathers would be of interest.

Overall, this study documents *P*. *huitzilopotchlii* prevalence and feather distribution on feathers from Anna’s and Black-chinned Hummingbirds, highlights the benefits of TSEM imaging for evaluating live mites on avian feathers, and provides a foundation for future hummingbird-mite relationship studies.
